# Synthesis and characterisation of bio-derived furan-based polyamides copolymers from dimethyl furan-2,5-dicarboxylate

**DOI:** 10.1039/d5ra09346e

**Published:** 2026-02-05

**Authors:** Zoe Paganelli, Lauri Välinen, Onsi Hanafi, Sami-Pekka Hirvonen, Hossein Baniasadi, Jukka Niskanen

**Affiliations:** a Polymer Synthesis Technology, School of Chemical Engineering, Aalto University Kemistintie 1 02150 Espoo Finland jukka.niskanen@aalto.fi; b Department of Chemistry, Faculty of Science, University of Helsinki 00014 Helsinki Finland

## Abstract

Polymers and plastics play integral roles in everyday life due to their versatility and durability. Among them, polyamides are particularly valued for their excellent properties and broad range of applications. The study explores the synthesis and characterisation of bio-derived polymers copolymers. Specifically, polyamides and copolyamides have been successfully synthesised from dimethyl furan-2,5-dicarboxylate and two diamines, hexamethylene diamine and 1,10-decanediamine, by employing a new three-step method. Key findings include the successful polymerisation of polyamides and copolyamides with a molecular weight up to 26 900 g mol^−1^ while demonstrating robust thermal stability, withstanding up to 403.7 °C. In addition, the mechanical properties, such as elastic modulus, of these amorphous polyamides and copolyamides were found to be comparable to commercial polyamides. Notably, the variation of molar ratios of diamines greatly influences the final properties of the polyamides and copolyamides, offering insight into customising mechanical, thermal, and physical qualities of the polymer. This research contributes significantly to the advancement of sustainable polymer solutions, positioning bio-derived polyamides as viable substitutes to mitigate reliance on fossil fuels while enhancing environmental sustainability.

## Abstract 2

Bio-derived polyamides and copolyamides are synthesised utilising a furan-based diacid, effectively tuning their characteristics depending on diamine molar ratio.

## Graphical abstract

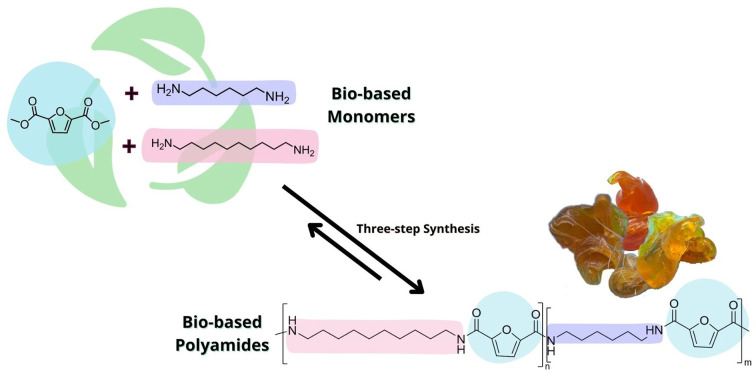

## 1 Introduction

Polyamides (PA) are widely used in demanding applications ranging from textile fibres to automotive and biomedical applications to electronics. As an example, polyamides poly(caprolactam) (PA6), poly(butylene adipamide) (PA6,6), poly(hexamethylene sebacamide) (PA610), and polylaurolactam (PA12) are commonly used as fibres and engineering plastics.^1–5^ It is estimated that the growing market value of polyamides will reach 53 billion USD by 2028.^2^ In addition, the demand for PAs is estimated to increase by 34.4% and exceed 11 Mt annually in 2025, compared to 2018.^5^

Aliphatic PAs, commonly known under the trade name nylons, are considered engineering polymers due to their excellent properties, including mechanical strength, thermal stability, and chemical resistance.^6^ However, aliphatic PAs have a notable disadvantage: their sensitivity to moisture, which adversely affects stability and mechanical properties.^2^ In contrast, aramids are fully aromatic polyamides known as high-performance polymers.^2,7^ The presence of aromatic moieties in the polymer chains contributes to their superior properties, including reduced moisture uptake, higher glass transition temperatures (*T*_g_) and melting temperatures (*T*_m_), and improved mechanical performance at elevated temperatures. However, aramids are challenging to melt-processes as they start to decompose before melting.^8,9^ Bridging the gap between aliphatic and aromatic polyamides, polyphthalamides (PPAs) are semiaromatic polyamides, offering exceptional mechanical and thermal stability as well as chemical resistance, while allowing for melt-processing.

Polyamide and polyphthalamide production is highly dependent on fossil resources, which results in environmental, social, and human health impacts. Hence, there is a demand to move towards sustainable and bio-based alternatives.^1,10–13^ The use of biomass as a feedstock to synthesise bio-based polymers is needed to decrease societal dependency on fossil sources while obtaining more sustainable products.^1,11,14–16^ According to a recent report by Plastics Europe,^17^ biomass-synthesised polymers can reduce CO_2_ emissions and act as carbon storage if coupled with a long life cycle. In 2024, European plastics production was around 55 Mt, with bio-based polymers accounting for about 1%.^18^ For comparison, the world plastics production was 431 Mt in 2024, of which 0.6% was bio-based plastics. However, the high availability of biomass feedstock is a significant driver for the development of bio-derived polymers and their future commercialisation. Although various biomass feedstocks are available for producing bio-based or semi-bio-based polyamides, poly(decamethylene terephthalamide) (PA10T) remains the only commercially available partially bio-based polyphthalamide.^2^ Overall, the use of biomass as feedstock to synthesise bio-based polymers represents a valid option to decrease dependence on fossil sources while obtaining more sustainable products.^1,11,14–16^

One of the bio-based monomers that sparks more interest is 2,5-furandicarboxylic acid (FDCA). This bio-derived monomer is commonly produced through the oxidation of 5-(hydroxymethyl) furfural (HMF), obtained from the degradation of a variety of biomass to glucose.^2,19–21^ Alternative routes for the production of FDCA and its derivatives use galactaric acid, derived from the oxidation of galactose, instead of HMF as a substrate.^22–24^ Due to its biodegradability, carbon neutrality, and physicochemical properties, FDCA was identified as one of the twelve most valuable bioplatform compounds.^1,25–29^ Furthermore, it has gained interest as a substitute for terephthalic acid in the synthesis of polyesters such as poly(ethylene furanoate) (PEF), a potential substitute for poly(ethylene terephthalate) (PET).^1,16,22,23,30^ FDCA can also be used to synthesise semi-aromatic polyamides, specifically furan-aliphatic polyamides (PAF).^1,2^ However, while the synthesis of PEF has almost reached commercialisationDutch company Avantium on the verge of commercialising Releaf^®^,^31^ some major drawbacks in the synthesis of polyamides from FDCA hinder their development. First, the low reactivity and poor thermal stability of FDCA impair polymerisation and limit the synthesis temperature of polyamides.^29^ Furthermore, the utilisation of FDCA generally leads to low molecular weight polymers due to side reactions hindering the growth of the polymer. Specifically, decarboxylation of FDCA and *N*-methylation have been reported to be the main cause of low molecular weight when the ammonium salt route is used as a synthesis method.^1,2,29,32,33^ Consequently, other routes were experimented with, including the use of solvents to various extents, and enzymatic polymerisation.^7,33,34^ Some of the shortcomings of these synthesis methods include costly purification methods and the impracticality of scaling-up.^2^

For these reasons, FDCA derivatives are of interest.^1,2,26,30,35–38^ In particular, dimethyl furan-2,5-dicarboxylate (DMFDC), which is the esterified derivative of FDCA, is more reactive and polymerisations has fewer side reactions. Xie *et al.*,^39^ successfully synthesised polyamides using DMFDC and different aliphatic (C4, C5, C6, C8, C12) diamines. In their work, Xie *et al.*^39^ have obtained polymers with molecular weight (*M*_w_) between 29 000 g mol^−1^ and 65 000 g mol^−1^. The process used consisted of a multi-step process where 1,5,7-triazabicyclo[4.4.0]dec-5-ene (TBD) dissolved in THF was used as the catalyst. Kamran *et al.*,^2^ describe a two-step melt polymerisation process in a thin film reactor using DMFDC and 1,6-hexamethylenediamine (HMDA). Specifically, the first step of the synthesis was carried out at a relatively low temperature (65 °C) under an inert atmosphere, which promoted oligomerisation while limiting *N*-methylation and promoting the removal of some of the side products. The polymerisation was then conducted under reduced pressure at 230 °C. This method yielded amorphous polymers with *M*_w_ ranging from 10 000 to 43 000 g mol^−1^, depending on catalyst load and diamine excess. In a following research, Kamran *et al.*^40^ synthesised polyamides from combining DMFDC and diamines with various chain lengths (C4, C6, C8, C10), obtaining high molecular weight polymers (*M*_w_ 23 000–36 000 g mol^−1^).

While FDCA- and DMFDC-based polyamides have been reported, prior studies typically examine single diamines without exploring compositional copolymer series or achieving relatively high molecular weight. The present study reports the systematic copolymerisation of DMFDC with 1,6-hexamethylenediamine and 1,10-decanediamine to obtain fully bio-based furan-aliphatic polyamides.^41,42^ The diamines were combined at varied feed ratios, which allows for the control and tuning of the thermal and mechanical properties of the obtained copolyamides. The reaction yielded polyamides and copolyamides with the glass transition temperature varying from 70 to 120 °C, and a Young's modulus from 1.3 to 2.6 GPa. In addition, the degradation temperature (*T*_d_) could be shifted to a higher temperature, from 390 to 404 °C, by copolymerisation. The resulting copolyamides were characterised by Fourier-transform infrared spectroscopy (FTIR), nuclear magnetic resonance (^1^H NMR), size exclusion chromatography (SEC), differential scanning calorimetry (DSC), thermo gravimetrical analysis (TGA), and dynamic mechanical analysis (DMA) to determine the influence of composition on the thermal and mechanical properties, but also the molecular weight of the copolyamides. In addition, the process was performed using a solvent-free method.

## 2 Experimental

### 2.1 Materials

Dimethyl furan-2,5-dicarboxylate (DMFDC, purity 98%) and hexafluoroisopropanol-D_2_ (≥99 atom% D) were obtained from AA Blocks Inc. (U.S.A.) and Eurisotop, respectively. 1,10-decanediamine (purity > 98%) was purchased from Tokyo Chemical Industry (TCI). 1,1,1,3,3,3- hexafluoro-2-propanol (HFIP, distilled) was acquired from Fluorochem. Poly(methyl methacrylate) (PMMA) standards were obtained from Polymer Standards Service, now Agilent. 1,6-hexamethylenediamine (HMDA, purity 98%), sodium hypophosphite monohydrate (purity ≥99%), and potassium trifluoroacetate (purity 98%) were procured from Sigma-Aldrich.

### 2.2 Synthesis of furan-based polyamides

The polycondensation parameters were adapted from the procedure reported by Kamran *et al.*^2^ The polycondensation of DMFDC with HMDA and 1,10-decanediamine is shown in Fig. 2. The polymerisation consisted of a three-step procedure. As an example, the copolyamide with 50 mol% HMDA and 50 mol% 1,10-decanediamine was synthesised as follows: DMFDC (30 g, 0.16 mol), along with HMDA (14 g, 0.08 mol) and 1,10-decanediamine (10.4 g, 0.09 mol), were charged into a 250 mL round-bottom flask together with sodium hypophosphite monohydrate (0.5 wt% of DMFDC). A 10 mol% excess of HMDA over the moles of DMFDC was used in the copolymerisations and the synthesis of PAF6. An excess of HMDA was used since it was observed to sublimate from the reaction during the first stage of the polymerisation. In the synthesis of PAF10 1 : 1 molar ratio of DMFDC and 1,10-decanediamine was used. Sodium hypophosphite monohydrate was used as the catalyst.

The flask was attached to a distillation set-up connected to a Schlenk line and purged with nitrogen for 10 minutes at room temperature while stirred with a magnet. The set-up was then sealed, and subsequently heated to 240 °C in steps. Firstly, the temperature was raised to 120 °C (200 rpm) for 20 minutes to allow the reactants to melt, start the formation of oligomers, and remove some of the side product (methanol). Secondly, the temperature was raised to 175 °C (100 rpm) for 1 hour and 30 minutes. This step led to the distillation of most of the methanol released from the DMFDC during transesterification. Lastly, after the methanol was removed from the collecting flask, the temperature of the reaction flask was increased to 240 °C (100 rpm), and the pressure decreased to 3 mbar. The system was kept in this condition for 24 hours. The entire procedure lasted 26 hours, including the time needed for the temperature increase between steps. Due to a significant increase in the viscosity, the magnetic stirring stopped at the end of the last step. The polymers were collected at 200 °C. The rest of the polyamides and copolyamides were synthesised similarly, and the experimental details are presented in Table 1. The samples were labelled as follows: poly(hexamethylene-furandicarboxylamide) (PAF6); poly(decamethylene-hexamethylene-furandicarboxylamide)s, depending on the HMDA and 1,10-decanediamine ratio in the polyamides, (PAF10,6_95-5, PAF10,6_75-25, or PAF10,6_50-50); and poly(decamethylene-furandicarboxylamide) (PAF10). ^1^H NMR (400 MHz HFIP-D_2_): 1.40 (–C*H*_2_–), 1.48 (–C*H*_2_–), 1.70 (–C*H*_2_–N), 3.48 (–C*H*_2_–CH_2_–N), and 7.14 (–C*H*=) ppm. The scalability of the process is yet to be determined and will be part of future research.

**Table 1:** Summary of the monomers and their ratios used in the polymerisations, including the molar ratio of 1,10-decanediamine in the final polymer determined from ^1^H NMR

Sample name	DMFDC			HMDA				1,10-Decanediamine			
	g	mol	mol ratio	g	mol	mol ratio	^1^H NMR ratio	g	mol	mol ratio	^1^H NMR ratio
PAF10	30	0.16	1	0	0	0	0	28.1	0.16	1	1
PA10,6_95-5	30	0.16	1	1.04	0.02	0.15	0.8	26.7	0.15	0.95	0.92
PAF10,6_75-25	30	0.16	1	5.21	0.06	0.35	0.30	21.1	0.12	0.75	0.70
PAF10,6_50-50	30	0.16	1	10.4	0.1	0.6	0.56	14	0.08	0.5	0.44
PAF6	30	0.16	1	20.8	0.18	1.1	1	0	0	0	0

**Fig. 1:**
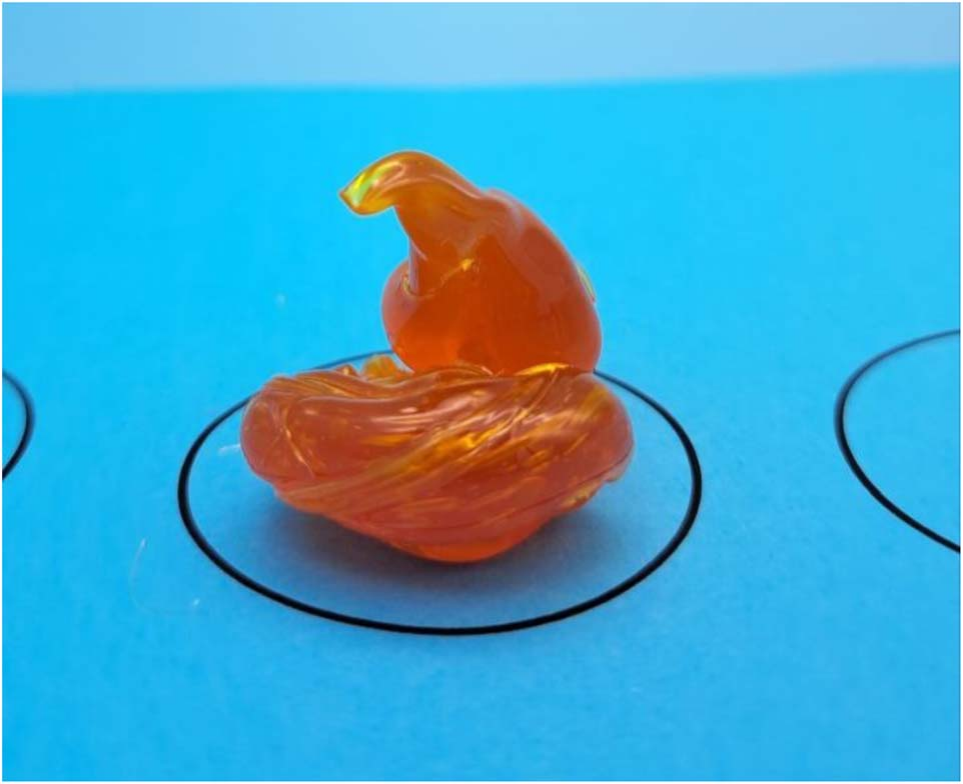
Picture of poly(decamethylene-hexamethylene-furandicarboxylamide) (PAF10,6_50-50).

Fig. 1 shows the PAF10,6_50-50 sample. Photographs of all synthesised polyamides and copolyamides are provided in the SI (Fig. S1).

**Fig. 2:**
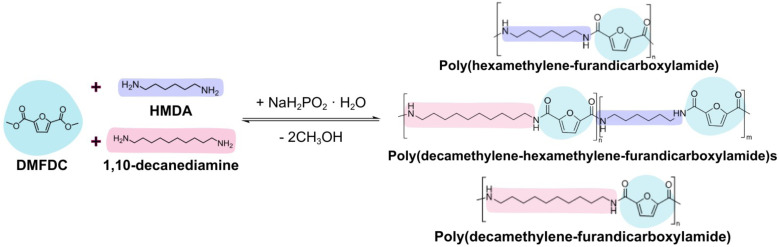
Polycondensation of DMFDC and HMDA/1,10-decanediamine.

### 2.3 Characterisation

#### 2.3.1 Molecular structure

Fourier-transform infrared spectroscopy (FTIR) analysis of the obtained polyamides and copolyamides was performed using a PerkinElmer FTIR instrument equipped with an ATR device. The scans were performed in transmittance mode in a wavenumber range of 4000 cm^−1^ to 450 cm^−1^.

Nuclear magnetic resonance (^1^H NMR) spectra were collected with a Bruker AVIII400 (400 MHz) spectrometer. The dissolution of the obtained polyamides was carried out using a concentration of 10 mg mL^−1^ in 1,1,1,3,3,3-hexafluoro-2-propanol-D2. The residual solvent signal at 4.40 ppm from HFIP was used as the reference.^43^

#### 2.3.2 Molecular weight

Size exclusion chromatography (SEC) was used to determine the number-average molecular weight (*M*_n_), the weight-average molecular weight (*M*_w_) and the dispersity (*Đ*). The system utilised consisted of Waters 515 HPLC pump, Waters 717 plus Autosampler and Waters 2410 Differential Refractometer. The eluent used was HFIP (recycled by distillation), with the addition of 3 g L^−1^ of potassium trifluoroacetate. Flow rate was 0.400 mL min^−1^, and the column (Agilent HFIP gel 300 × 7.5 mm) was thermostated at 45 °C. All samples were filtered using 13 mm 0.2 µm PTFE syringe filters (Fisherbrand) prior to measurement. PMMA standards having *M*_p_ between 602 and 1 510 000 were used for calibration. Data were analysed using Omnisec 4.7 software.

#### 2.3.3 Thermal properties

Differential scanning calorimetry (DSC) was performed to determine the thermal properties of the polyamides and copolyamides, using a TA Instruments Discovery DSC 250. Considering the traces, only the glass transition temperature (*T*_g_) was evaluated. Specifically, 5 mg of sample was sealed within a Tzero aluminium pan and subjected to two heating and cooling cycles under a nitrogen flow of 50 mL min^−1^. The samples were equilibrated at −30 °C, then heated up to 280 °C and finally cooled to 0 °C, both at a rate of 10 °C min^−1^. The second heating and cooling cycle was used to extract data for the thermal transition temperatures.

The thermal decomposition of the polyamides and copolyamides was investigated under a nitrogen atmosphere using thermogravimetric analysis (TGA, TA Instruments model TGA 5500). The samples were initially equilibrated at 30 °C before heating to 800 °C at a rate of 10 °C min^−1^. Thermal decomposition temperature (*T*_d_), was determined at 5% mass loss. The maximum decomposition temperature (*T*_max_), and the residue at 800 °C were also extracted and analysed from the TGA and DTG plots and are presented as SI.

#### 2.3.4 Mechanical analysis

The dynamic mechanical properties were analysed using a TA Instruments device model Q800 under a multi-frequency-strain procedure mode. Films suitable for the analysis were obtained by hot pressing the polyamides and copolyamides. Specifically, the polymer was pressed for 10 minutes at 150 °C and 150 kN. Afterwards, strip-shaped samples were made by punching the films. Strip-shaped samples were clamped into the device, while the preload, strain, and frequency were set at 1 N, 0.5%, and 1 Hz, respectively. The temperature was set to increase from 30 °C to 145 °C at a heating rate of 5 °C min^−1^. The plots of storage modulus (*E*′), loss modulus (*E*″), and loss factor (tan *δ*) *versus* temperature were analysed. Glass transition temperature (*T*_g_) and Young's Modulus were extrapolated from the tan *δ* plots and the *E*′ plots, respectively. Due to constraints on available sample quantities and the challenges in obtaining samples suitable for DMA, it was not possible to carry out all tests in triplicate. Despite this limitation, rigorous attention was given to experimental procedures and data collection to maintain the integrity of the results. Future work will aim to obtain scale-up to adhere more closely to standard practices in addition to further analysing the polyamides and copolyamides.

## 3 Results and discussion

### 3.1 Structure characterisation

**Fig. 3:**
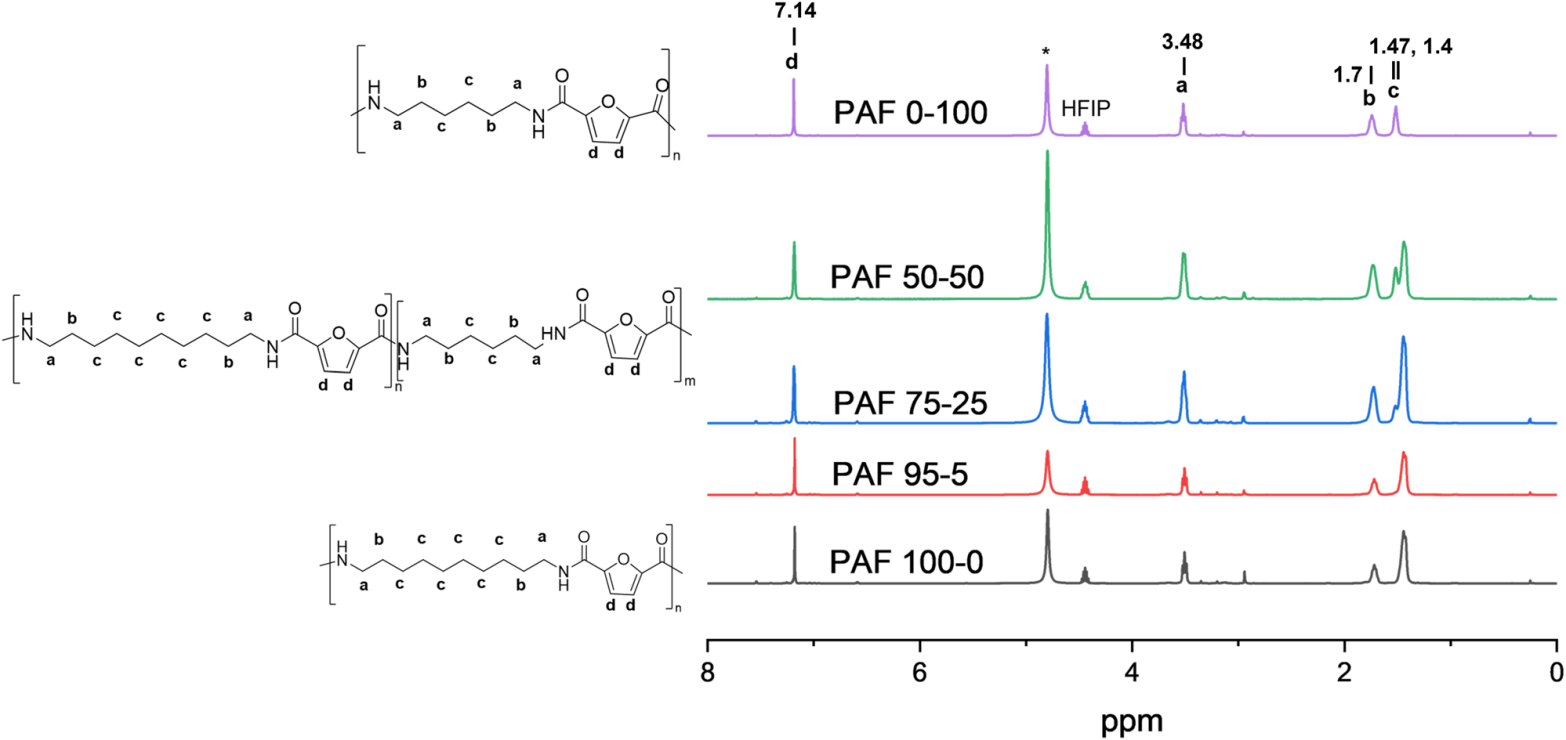
^1^H NMR spectra of the synthesised polyamides and copolyamides. * is used to mark the water peak.

**Fig. 4:**
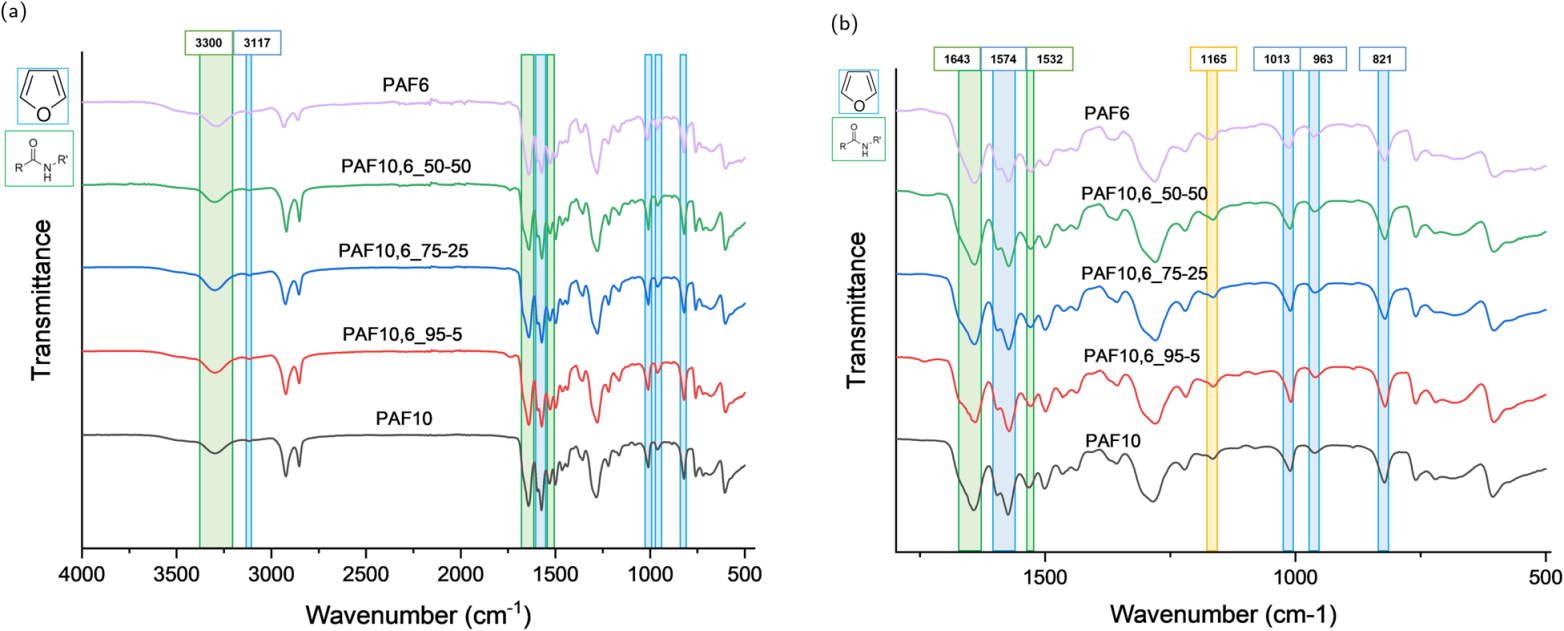
FTIR spectra of the synthesised polyamides and copolyamides, highlighting characteristic peaks of the furan ring and amide groups. The figure on the left (a) portrays the full FTIR spectra, while the one on the right (b) focuses on the wavenumbers between 1750 cm^−1^ and 500 cm^−1^.

The chemical structure of the synthesised polyamides and copolyamides was characterised by ^1^H NMR (Fig. 3 and S2–S6), and FTIR spectroscopy (Fig. 4). The peaks present in the spectra are comparable to those in the literature, and are proof of successful polymerisation.^29,38–40^ It should be noted that the peaks are shifted compared to what has been reported, due to the use of different solvents (DMSO-d_6_, CDCl_3_/HFIP).

In the ^1^H NMR spectrum for PAF10,6_50-50, the characteristic signal of the protons belonging to the furan ring is observed at 7.14 ppm, while the protons from the aliphatic chain are observed at 1.40, 1.48, and 1.70 ppm (Fig. S3). The signal from the methylene group next to the amide group is observed at 3.48 ppm. The signals from the methylene groups are observed to move upfield, *i.e.* 1.70 to 1.68 ppm and 1.47 to 1.40 ppm, with increasing 1,10-decanediamine content in the polyamides and copolyamides (Fig. S4). A similar observation can be made for the protons from the furan ring, where the signal shifts upfield from 7.14 to 7.13 ppm (Fig. S5). In addition, the signal from the methylene groups in decanediamine (1.40 ppm) is slightly more upfield than the signals from hexanediamine (1.47 ppm). This allowed us to estimate the final content of the diamine monomers in the final copolyamides from the spectra by comparing the signals at 1.70 ppm (4 protons originating from both HDMA and 1,10-decanediamine) and 1.40 ppm (12 protons from 1,10-decanediamine): PAF10,6_50-50 had 44% decanediamine and PAF10,6_75-25 and PAF10,6_95-5 had 70% and 92%, respectively (Table 1). The integrals used in the calculation are presented as SI in Fig. S6.

Fig. 4 shows the FTIR spectra of the synthesised polyamides and copolyamides, which show the typical absorbance bands of furan-based amides.^2,7,38–40^ Characteristic absorbance bands for the aromatic ring can be observed at around 3100 cm^−1^ (C–H stretching) between 1500 and 1600 cm^−1^ (C=C stretching vibration), around 1020 cm^−1^ (C–O–C), and at 960 cm^−1^ and 820 cm^−1^ (C–H bending). In particular, observable absorbance bands associated with the furan ring were observed at 3117 cm^−1^ (C–H stretching), 1574 cm^−1^ (C=C), 1013 cm^−1^ (C–O–C), and 963 cm^−1^ and 821 cm^−1^ (C–H bending). Regarding amide groups, the N–H stretching vibration of both polyamides and copolyamides was found as a broad absorbance band around 3300 cm^−1^. The absorbance band of amide I could be seen around 1643 cm^−1^, whereas the absorbance band of amide II was assigned to 1532 cm^−1^. The peak at 1165 cm^−1^, highlighted in yellow in Fig. 4b, could be attributed to both C–N stretching from secondary amides or C–O stretching related to ester groups. This peak could signify a partial remaining of unreacted monomer in the polyamides and copolyamides. However, no absorbance bands could be found at other wavenumbers that could be associated with the monomers, such as the stretching belonging to the free C=O from the carboxylic group of the acid, confirming the effective conversion of the monomers.^7^

Following the synthesis, the polyamides and copolyamides showed different shades of colour, which was observed to differ according to the increasing concentration of HMDA and vary from yellow to a warmer orange (Fig. S1). The yellow-orange hue of the polyamides and copolyamides is believed to be related to the formation of oligo-enimine structures formed during the polymerisation.^2,29,44,45^ Further optimisation of the colour of the polyamides and copolyamides has been considered to be future research, as it is influenced by *e.g.* reactor set-up, conditions, and the choice of catalyst. In this study, sodium hypophosphite monohydrate was chosen as the catalyst since it is used extensively in the synthesis of PAs.^46^ However, we acknowledge that further research on the performance of different catalysts on the polymerisation is needed and will be part of future research.

### 3.2 Molecular weight

The molecular weights and dispersities of the polyamides and copolyamides are presented in Table 2. The *M*_w_ ranged from over 10 000 g mol^−1^ to almost 27 000 g mol^−1^. The results obtained are comparable to what has been reported by other researchers, where *M*_w_ of polyamides containing furan dicarboxylic acid ranged from 3000 to 65 500 g mol^−1^.^2,29,37–39^ Notably, the *M*_w_ increased with increased amount of HMDA in the feed, resulting in PAF10,6_50-50 being the polymer with the highest *M*_w_ (26 900 g mol^−1^ and *Đ* 2.7), while PAF10 had the lowest *M*_w_ (10 300 g mol^−1^ and *Đ* 2.8). This can be explained by the evaporation of the diamine during the polymerisation process. For instance, HMDA can evaporate more easily from the polymer melt due to its lower boiling point (HMDA *b*_p_ 204 °C at 1013 mbar, 1,10-decanediamine *b*_p_ 140 °C at 16 mbar). An excess of 10 mol% of HMDA, with respect to DMDCF, was used in the reactions to ensure all of the DMDCF reacted, and that the species to be removed during polymerisation was the amine and not DMFDC. However, the high and increasing viscosity of the polymer melt during the melt polymerisation limited its stirring, which affected the diffusion and evaporation of the HMDA in the late stages of polymerisation. An excess of HMDA has been shown to lower the melt viscosity by disrupting the interchain interactions.^2^ Hence, a higher concentration of HMDA during polymerisation led to lower melt viscosity and more effective stirring, resulting in improved molecular weight polyamides and copolyamides.^47^

**Table 2:** The molecular weight, dispersity (*Đ*), glass transition temperature (*T*_g_) from both DSC and DMA analysis, and thermal degradation temperature (*T*_d_) of the polyamides

Sample	*M*_n_ (g mol^−1^)	*M*_w_ (g mol^−1^)	*Đ*	*T*_g_ a (°C)	*T*_g_ b (°C)	*T*_d_ (°C)	Young's modulus (MPa)
PAF10	3700	10 300	2.8	70.0	n.a.	403.7	—
PAF10,6_95-5	3900	14 900	3.8	80.1	n.a.	403.3	—
PAF10,6_75-25	6800	21 700	3.2	92.7	126.7	398.8	1507
PAF10,6_50-50	10 100	26 900	2.7	101.8	135.3	395.5	1367
PAF6	9900	26 900	2.7	119.2	140.6	389.6	2598

a DSC analysis.

b DMA analysis.

### 3.3 Thermal and mechanical properties

**Fig. 5:**
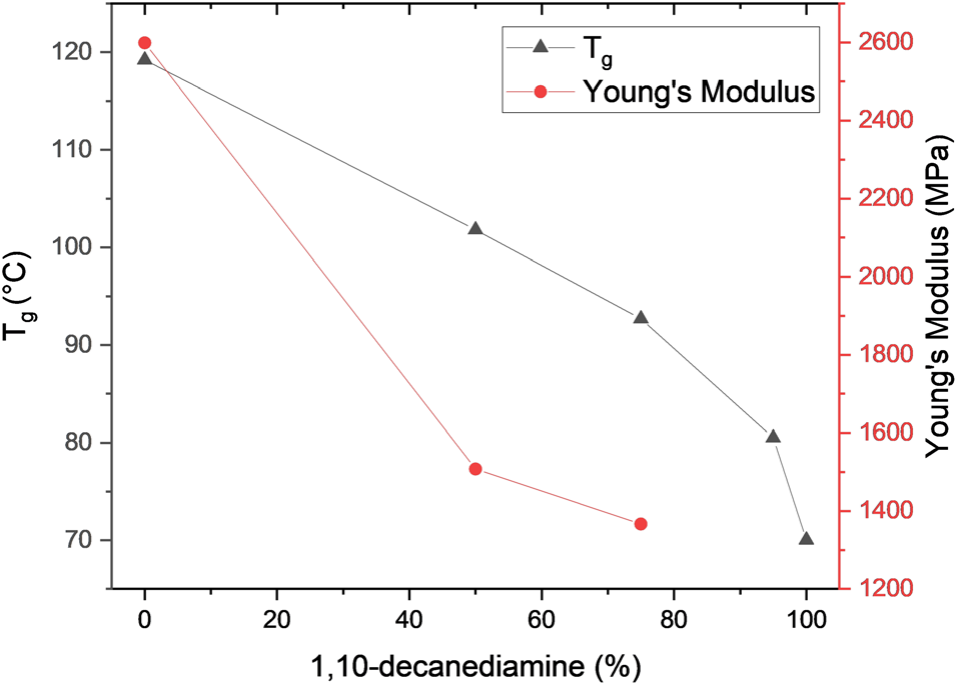
Plot showing the variation of glass transition temperature, *T*_g_, from DSC and the Young's modulus, as a function of the 1,10-decanediamine content.

Only one transition, corresponding to the glass transition temperature (*T*_g_), can be observed in the DSC traces of the polyamides (Fig. S7). The *T*_g_ is observed to decrease with increasing 1,10-decanediamide content, as shown in Fig. 5. PAF10, the polyamide with 100% 1,10-decanediamide, had the lowest *T*_g_ of 70.0 °C. The observed *T*_g_s increased to up to 101.8 °C with increasing HMDA content in the copolyamides; for PAF10,6_95-5, PAF10,6_75-25, and PAF10,6_50-50, the *T*_g_s were 80.1, 92.7, and 101.8 °C, respectively (Table 2). The highest *T*_g_, 119 °C, was observed for PAF6, the polyamide with 100% HMDA.

The observed decrease in the *T*_g_ is related to the length of the aliphatic chain of the diamine. Longer aliphatic chains in polyamides are known to lower the *T*_g_ of polyamides: a *T*_g_ of 80 °C is reported for PA6,6, whereas for PA6,10 the *T*_g_ is 65 °C, and for PA12 is 58 °C.^48^ The lower carbon number in HDMA leads to a higher hydrogen bond density and a stiffer structure, which increases the *T*_g_.^2,7,39,49,50^ Consequently, it is possible to tune the *T*_g_ by changing the molar ratio of HMDA and 1,10-decanediamine.

Polyamides containing furan rings have been shown to be amorphous polymers.^2,7,38,39,45,51^ Indeed, the thermograms do not show typical crystallisation nor melting usually observed for polyamides, neither in the heating, nor in the cooling cycles. The angle of the carboxyl groups on the furan ring hinders the bulk crystallisation of the polyamides by weakening interchain hydrogen bonds between amides and carbonyl groups. Specifically, the difference in the angle between the carboxylic acid groups attached to the furan ring (129.4°) *versus* the *para*-substituted benzene in terephthalic acid (180°) reduces the linearity of the chain, altering crystallisation kinetics and thereby hindering the crystallisation of the furandicarboxamide moiety.^7,51^ For comparison, a polyamide made from terephthalic acid and HMDA has a melting temperature (*T*_m_) of 267 °C, while no *T*_m_ was observed for PAF6.^52^

PAF10 displayed the highest thermal stability, with an onset decomposition temperature (*T*_d_) of 403.7 °C, while PAF6 had the lowest *T*_d_ of 389.6 °C (Table 2 and Fig. S8). The *T*_d_ of the copolyamides decreased with HMDA content. The observed *T*_d_s are in the range of the typical semi-aromatic polyamides.^45^ For comparison, PA6,6 starts to decompose at 359 °C, while aramides and polyphthalamides are more stable, degrading at over 400 °C.^21,53,54^

Different thermal degradation mechanisms of polyamides have been documented depending on the length of the aliphatic chain.^9^ The thermal degradation of short chains is characterised by cyclisation and C–N bond cleavage, whereas β-CH hydrogen transfer predominantly drives the degradation of long chains. In semi-aromatic PAs, the degradation mechanisms at low temperatures include β-CH transfer, C–N bond cleavage, and cyclisation, while at higher temperatures radical scission becomes predominant.^2,9^ The residue at 800 °C increased from 1.9% to 11.9% with increasing HMDA content, indicating differences in the degradation processes of the polyamides depending on the copolyamide composition (SI, Table S1).

The degradation of the polyamides is also linked to the degradation of the furan moieties.^7,55^ FDCA was observed to start degrading by undergoing decarboxylation at 264 °C (SI, Fig. S7) and even lower temperatures (180 °C) for the degradation of FDCA have been reported.^55^ The low degradation temperature of the carboxylic acid not only influences the polymerisation process but also affects the thermal stability of the polymers. DMFDC exhibits better thermal stability and is therefore a more suitable monomer to use in the synthesis of polyamides.^55^ To summarise, the use of a furan-containing carboxylic acid imparts good thermal stability to the polyamides and copolyamides while maintaining low processing temperatures compared to aromatic polyamides. Hence, they are suitable for various processing methods, such as injection moulding, melt spinning, or 3D printing.

In this study, the obtained plots showing the storage modulus (*E*′), loss modulus (*E*″), and loss factor (tan *δ*) are shown in SI in Fig. S10.

Unfortunately, it was not possible to analyse PAF10 and PAF10,6_95-5 due to their brittleness. Consequently, the mechanical analysis mainly aims to demonstrate the variation of properties related to differences in monomer composition.

As observed in the DSC measurements, the *T*_g_ obtained from the tan *δ* plot (Fig. S10c), decreases with increasing 1,10-decanediamine content in the polyamides and copolyamides: 140.6 °C (PAF6), 135.3 °C (PAF10,6_50-50), and 126.7 °C (PAF10,6_75-25).

Considering the Young's moduli (*E*′), the polyamides become softer with increasing 1,10-decanediamine content. Specifically, the modulus decreases from 2598 MPa to 1507 MPa and 1367 MPa, for PAF6, PAF6,10_75-25, and PAF6,10_50-50, respectively (Fig. 5 and Table 2). For comparison, Young's moduli have been reported in the range of 1800 to 3800 MPa for PAF6.^39,40^ Young's moduli for the aliphatic polyamides PA6 and PA6,10 have been reported as 721 and 506 MPa.^48^ Xie *et al.* also reported a similar trend in decreasing moduli with longer aliphatic chain comonomers, connecting the phenomenon to the hydrogen bond density.^39^ Moreover, the rigid furan moiety in the furan-based polyamides increases the stiffness of the investigated polyamides and copolyamides, thereby increasing the Young's modulus when compared to aliphatic polyamides.

Molecular weight plays a complementary role in the observed Young's moduli. Increasing the molecular weight of a polymer can increase the Young's modulus and affect other properties of polymers. As discussed previously, increasing HMDA content yielded polyamides with higher *M*_w_; hence, the stiffness improved. A higher *M*_w_ is also linked to enhanced intermolecular interactions, which can positively affect the toughness of the material.^56^ Consequently, polymers with higher molecular weight display better film-forming properties, higher strength at break, and resistance to crack propagation. This, in addition to lower ability for energy dissipation given by flexible segments, is likely the reason behind the difficulty in obtaining samples suitable for the DMA analysis from PAF10 and PAF6,10_95-5.

## 4 Conclusions

This paper presents fully bio-based polyamides based on DMFDC, HMDA and 1,10-decanediamine using melt condensation polymerisation reaching *M*_w_ of 27 000 g mol^−1^. The *M*_w_ of the obtained polyamides was observed to depend on the HMDA content, as an increasing amount of HMDA yielded in polyamides with higher molecular weights. This is due to the possibility of evaporating HMDA during the polymerisation process, along with water, to drive the polymerisation to higher molecular weights. The comonomer content was confirmed to be similar to the feed ratios by ^1^H NMR: 92%, 70%, and 44% compared to the feed contents of 95%, 75%, and 50% of 1,10-decanediamine.

All of the obtained polyamides and copolyamides were amorphous due to the furan moieties. Hence, no melting nor crystallisation of the samples was observed in the DSC traces. The *T*_g_ was observed to decrease with increasing 1,10-decanediamine content from 102 °C (PAF10,6_50-50) to 70 °C (PAF10). For comparison, the *T*_g_ of PAF6, containing only HMDA, was 119 °C. Similar dependency of the monomer composition on the *T*_g_ was observed by DMA. In addition, the Young's modulus of the polyamides was observed to decrease with increasing 1,10-decanediamine content, as the Young's modulus decreased from 2598 MPa to 1507 MPa and 1367 MPa, for PAF6, PAF6,10_75-25, and PAF6,10_50-50, respectively. The thermal stability was also observed to increase with increasing 1,10-decanediamine content in the polyamides and copolyamides, as *T*_d_ increased from 389.6 °C (PAF6) to 403.7 °C (PAF10).

Both the thermal and mechanical properties of the copolyamides can be tuned by the ratio of the diamine monomers, HMDA and 1,10-decanediamine. The high thermal stability of the polyamides and copolyamides compared to the relatively low glass transition temperature underscores the possibility of using these polymers for various processes, such as moulding, spinning, and 3D printing. Depending on the desired characteristics, these novel polyamides and copolyamides could be used in a variety of applications, for example, fibres, sensors, and automotive parts, although further investigation is necessary to scale-up the process and commercialise these polyamides as products. Specifically, lower oligomerisation and polymerisation temperatures should be investigated while implementing a set-up that allows for higher vacuum to be reached, to help synthesise higher molecular weight polyamides and copolyamides.

In addition, in light of the ongoing climate crisis and the detrimental effects of fossil fuel exploitation, transitioning to renewable solutions is vital. Implementing a thorough life cycle assessment (LCA) of newly developed bio-based materials is crucial to ensure their sustainability and prevent green-washing. By developing new bio-based polymers and assessing the environmental impact throughout the entire lifecycle, truly sustainable polymers can be developed that contribute positively to society's future.

## Author contributions

Zoe Paganelli: conceptualisation, methodology, formal analysis, investigation, writing – original draft, review & editing, visualisation, supervision, project administration. Lauri Välinen: formal analysis. Hanafi Onsi: formal analysis. Sami-Pekka Hirvonen: formal analysis; Hossein Baniasadi: writing – review & editing, Jukka Niskanen: methodology, writing – review & editing, supervision, funding acquisition.

## Conflicts of interest

There are no conflicts to declare.

## Supplementary Material

RA-016-D5RA09346E-s001

## Data Availability

The data supporting the findings of this study are available within the article and its supplementary information (SI). Supplementary information is available. See DOI: https://doi.org/10.1039/d5ra09346e.

## References

[cit1] Huang W., Hu X., Zhai J., Zhu N., Guo K. (2020). Mater. Today Sustain..

[cit2] Kamran M., Davidson M. G., de Vos S., Tsanaktsis V., Yeniad B. (2022). Polym. Chem..

[cit3] Otaegi I., Aramburu N., Müller A. J., Guerrica-Echevarría G. (2018). Polymers.

[cit4] Baniasadi H., Trifol J., Ranta A., Seppälä J. (2021). Composites, Part B.

[cit5] Gupta N. K., Reif P., Palenicek P., Rose M. (2022). ACS Catal..

[cit6] Fink J. K. , in High Performance Polymers, Partially Aromatic Poly(amide)s, Elsevier, 2nd edn, 2014, ch. 12

[cit7] Luo K., Wang Y., Yu J., Zhu J., Hu Z. (2016). RSC Adv..

[cit8] Reglero Ruiz J. A., Trigo-López M., García F. C., García J. M. (2017). Polymers.

[cit9] Yang K., Liu Y., Zheng Z., Lu G., Tang Z., Chen X. (2022). Polym. Degrad. Stab..

[cit10] Jambeck J. R., Geyer R., Wilcox C., Siegler T. R., Andrady M. P. A., Narayan R., Law K. L. (2015). Science.

[cit11] Liu L., Xu M., Ye Y., Zhang B. (2022). Sci. Total Environ..

[cit12] Ardolino F., Palladini A. R., Arena U. (2023). Sustain. Prod. Consum..

[cit13] Dormer A., Finn D. P., Ward P., Cullen J. (2013). J. Clean. Prod..

[cit14] Baniasadi H., Madani Z., Mohan M., Vaara M., Lipponen S., Vapaavuori J., Seppälä J. V. (2023). ACS Appl. Mater. Interfaces.

[cit15] Gupta S. S., Mishra V., Mukherjee M. D., Saini P., Ranjan K. R. (2021). Int. J. Biol. Macromol..

[cit16] Zhu Y., Romain C., Williams C. K. (2016). Nature.

[cit17] Plastics Europe , The Circular Economy for Plastics—A European Analysis, Plastics Europe Technical Report, 2024

[cit18] Plastics Europe , Plastics The Fast Facts 2025, Plastics Europe Technical Report, 2025

[cit19] Hou Q., Qi X., Zhen M., Qian H., Nie Y., Bai C., Zhang S., Bai X., Ju M. (2021). Green Chem..

[cit20] Chheda J. N., Huber G. W., Dumesic J. A. (2007). Angew. Chem., Int. Ed..

[cit21] Zhu P., Shi M., Shen Z., Liao X., Chen Y. (2024). Chem. Sci..

[cit22] van Strien N., Niskanen J., Berghuis A., Pöhler H., Rautiainen S. (2024). ChemSusChem.

[cit23] Niskanen J., Mahlberg R., van Strien N., Rautiainen S., Kivilahti E., Koivuranta K., Anghelescu-Hakala A. (2024). ChemSusChem.

[cit24] Trapasso G., Annatelli M., Dalla Torre D., Aricò F. (2022). Green Chem..

[cit25] Rosenfeld C., Konnerth J., Sailer-Kronlachner W., Solt P., Rosenau T., van Herwijnen H. W. (2020). ChemSusChem.

[cit26] Mao L., Pan L., Ma B., He Y. (2022). J. Polym. Environ..

[cit27] Werpy T. and Petersen G., Top Value Added Chemicals from Biomass: Volume I—Results of Screening for Potential Candidates from Sugars and Synthesis Gas, Technical report, National renewable energy lab. (NREL), Golden, CO (United States), 2004

[cit28] Hwang D. K., Chung S., Kim S., Park J., Ryu J., Park J., Oh D. X., Jeon H., Koo J. M. (2023). Polym. Degrad. Stab..

[cit29] Cousin T., Galy J., Rousseau A., Dupuy J. (2018). J. Appl. Polym. Sci..

[cit30] Sousa A. F., Vilela C., Fonseca A. C., Matos M., Freire C. S. R., Gruter G.-J. M., Coelho J. F. J., Silvestre A. J. D. (2015). Polym. Chem..

[cit31] Fei X., Wang J., Zhang X., Jia Z., Jiang Y., Liu X. (2022). Polymers.

[cit32] Cao M., Zhang C., He B., Huang M., Jiang S. (2017). Macromol. Res..

[cit33] Jiang Y., Maniar D., Woortman A. J. J., Loos K. (2016). RSC Adv..

[cit34] da Fontoura C. M., Pistor V., Mauler R. S. (2019). Polimeros.

[cit35] Papadopoulos L., Klonos P. A., Kluge M., Zamboulis A., Terzopoulou Z., Kourtidou D., Magaziotis A., Chrissafis K., Kyritsis A., Bikiaris D. N., Robert T. (2021). Polym. Chem..

[cit36] Grosshardt O., Fehrenbacher U., Kowollik K., Tübke B., Dingenouts N., Wilhelm M. (2009). Chem.-Ing.-Tech..

[cit37] Cureton L. S. T., Napadensky E., Annunziato C., Scala J. J. L. (2017). J. Appl. Polym. Sci..

[cit38] Xie S., Yang J., Wang X., Yang J. (2022). Eur. Polym. J..

[cit39] Xie S., Yu D., Yao J., Wei Z., Wang X., Yang J. (2024). J. Polym. Environ..

[cit40] Kamran M., Davidson M. G., Tsanaktsis V., Van Berkel S., De Vos S. (2022). Eur. Polym. J..

[cit41] Dros A. B., Larue O., Reimond A., De Campo F., Pera-Titus M. (2015). Green Chem..

[cit42] Wang X., Gao S., Wang J., Xu S., Li H., Chen K., Ouyang P. (2021). Chin. J. Chem. Eng..

[cit43] Muljajew I., Erlebach A., Weber C., Buchheim J. R., Sierka M., Schubert U. S. (2020). Polym. Chem..

[cit44] Bandi S., Mehta S., Schiraldi D. A. (2005). Polym. Degrad. Stab..

[cit45] Shen T., Zhang B., Wang Y., Yang P., Li M., Hu R., Guo K., Chen K., Zhu N., Wang L., Zhu C., Ying H. (2022). Chem. Eng. J..

[cit46] Zheng W., McAuley K. B., Marchildon E. K., Yao K. Z. (2007). Can. J. Chem. Eng..

[cit47] Feng J., Yan D., Rong C., Yu L., Li J., Xin J., Lu X., Zhou Q., Wang Z., Wei Z. (2025). Green Chem..

[cit48] Nguyen P. H., Spoljaric S., Seppälä J. (2018). Eur. Polym. J..

[cit49] Maniar D., Hohmann K. F., Jiang Y., Woortman A. J., Dijken J. V., Loos K. (2018). ACS Omega.

[cit50] Yeh I.-C., Rinderspacher B. C., Andzelm J. W., Cureton L. T., La Scala J. (2014). Polymer.

[cit51] Wilsens C. H., Deshmukh Y. S., Noordover B. A., Rastogi S. (2014). Macromolecules.

[cit52] Stouten J., Wróblewska A. A., Grit G., Noordijk J., Gebben B., Meeusen-Wierts M. H. M., Bernaerts K. V. (2021). Polym. Chem..

[cit53] Ma Y., Zhang J., Cao X., Wu P., Ye G., Fu Y., Zhuang Y., Zhang A., Zheng K., Ma Y. (2021). Mater. Adv..

[cit54] Kurima A., Nguyen T. A., Kinashi K., Sakai W., Tsutsumi N. (2023). Polym. Degrad. Stab..

[cit55] Papageorgiou G. Z., Tsanaktsis V., Bikiaris D. N. (2014). Phys. Chem. Chem. Phys..

[cit56] Balani K. , Verma V., Agarwal A. and Narayan R., Biosurfaces: a Materials Science and Engineering Perspective, 2015, vol. 329

