# Supplementary material for: Synthesis and characterisation of bio-derived furan-based polyamides copolymers from dimethyl furan-2,5-dicarboxylate
Source: RSC Adv. 2026 Feb 5;16(8):7581–9. doi: 10.1039/d5ra09346e (PMC12875312; doi:10.1039/d5ra09346e)
Supplement: RA-016-D5RA09346E-s001 [file RA-016-D5RA09346E-s001.pdf]

Supplementary Information (SI) for RSC Advances.
This journal is © The Royal Society of Chemistry 2026

Supporting Information

Synthesis and characterisation of bio-derived furan-based polyamides copolymers from dimethyl furan-2,5-dicarboxylate

Zoe Paganelli,[a] Lauri Välinen,[a] Onsi Hanafi,[a] Sami-Pekka Hirvonen,[b] Hossein Baniasadi[a]
Jukka Niskanen[a]

Contents

1 Images of Polyamides and Copolyamides 2

2 Nuclear Magnetic Resonance 3

3 Differential Scanning Calorimetry 7

4 Thermogravimetric Analysis 7

5 Dynamic Mechanical Analysis 9

[a] *Polymer Synthesis Technology, School of Chemical Engineering, Aalto University, Kemistintie 1, 02150 Espoo, Finland. E-mail: jukka.niskanen@aalto.fi*

[b] *Department of Chemistry, Faculty of Science, University of Helsinki, 00014 Helsinki, Finland*

1 Images of Polyamides and Copolyamides

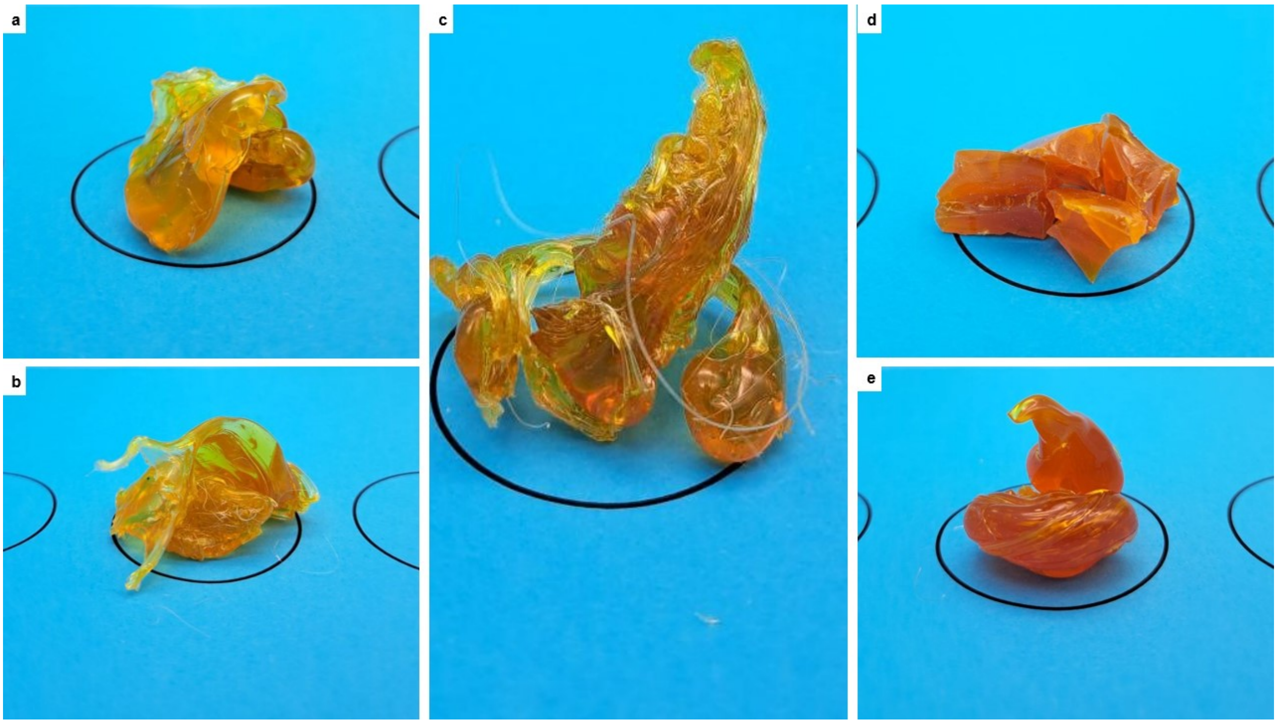

Figure S1: Figures of polyamides and copolyamides: a) PAF10, b) PAF10,6_75-25, c) PAF10,6_95-5, d) PAF6, e) PAF10,6_50-50.

2 Nuclear Magnetic Resonance

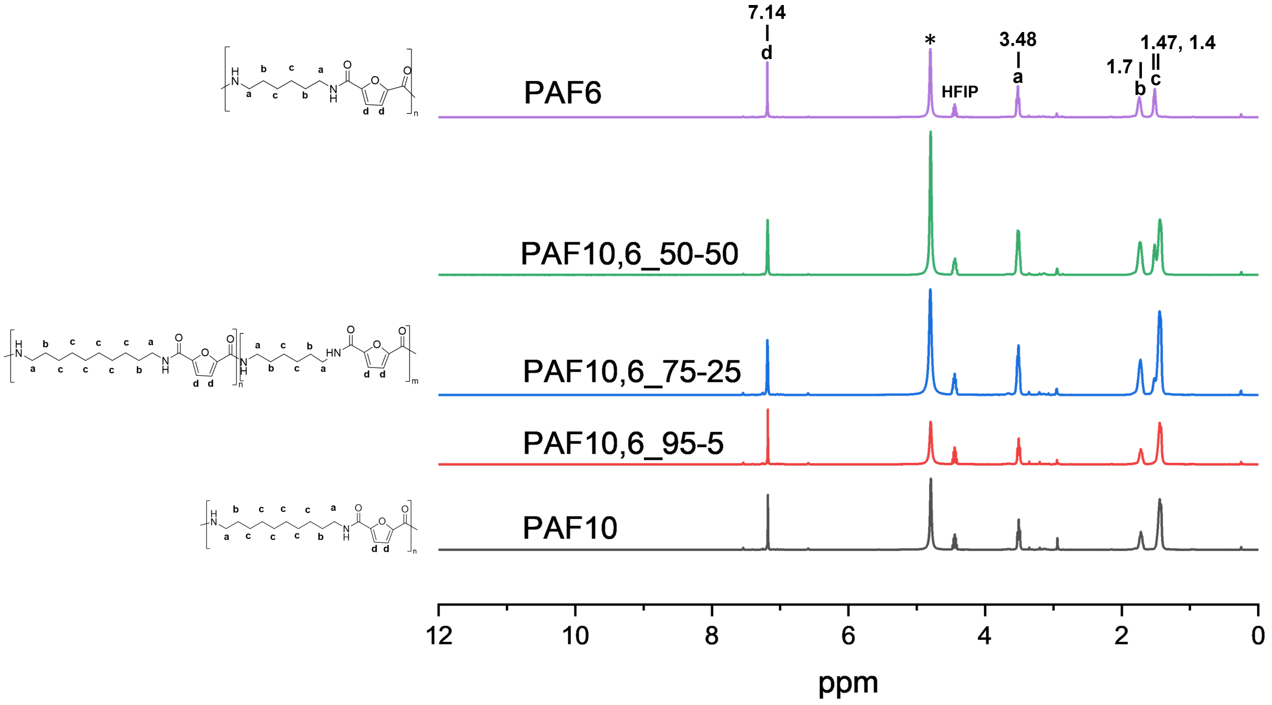

Figure S2: Full ^{1}H-NMR spectrum of the polyamides and copolyamides in HFIP-d_2.
$*$ is used to mark the water peak.

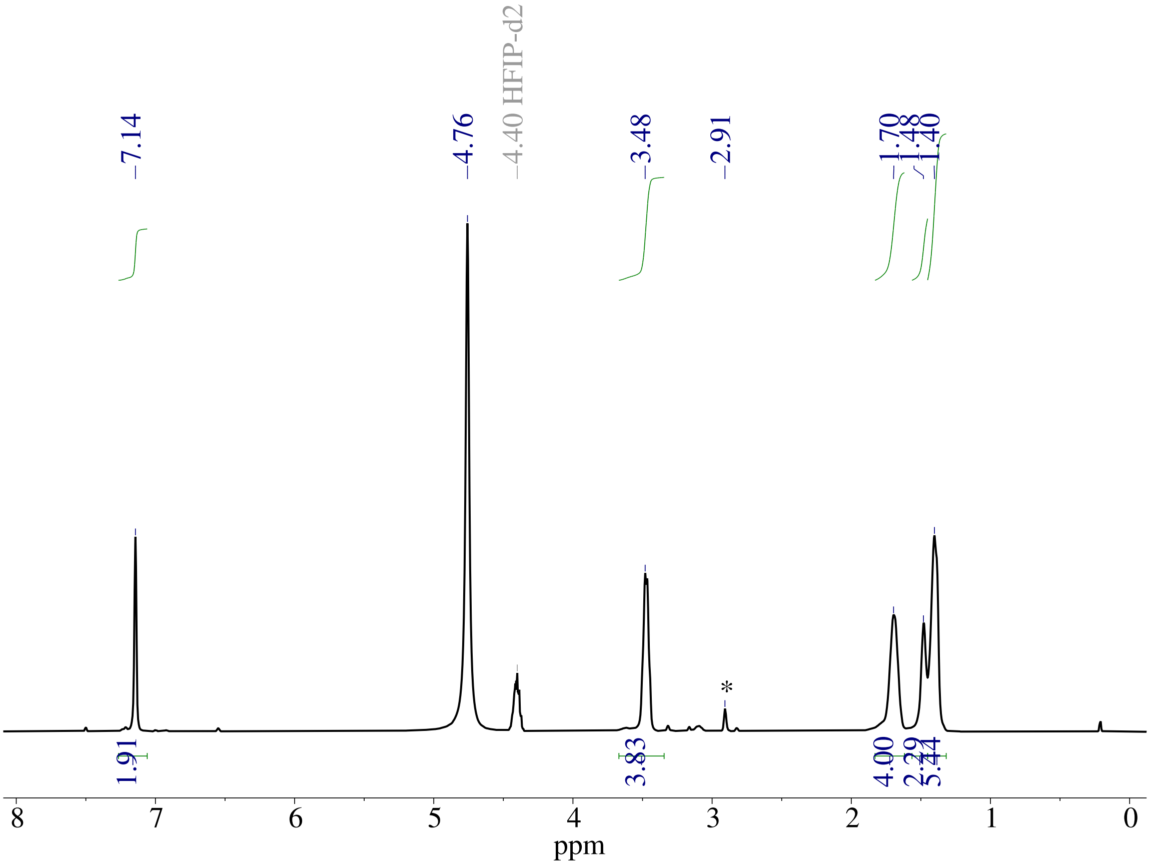

Figure S3: ^{1}H-NMR spectrum of PAF10,6_50-50 in HFIP-d_2.

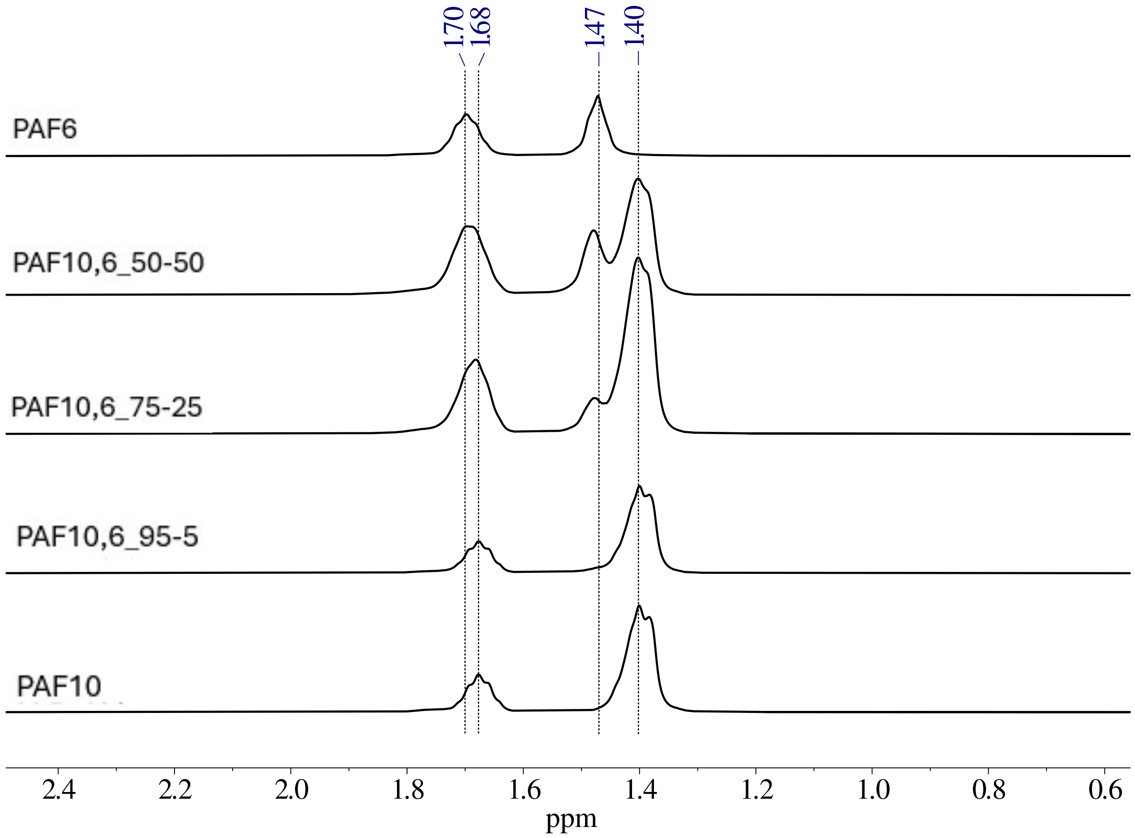

Figure S4: The alkane region of ^{1}H-NMR spectra of the polyamides, showing the upfield shift of the signals with increasing amount of decanediamine.

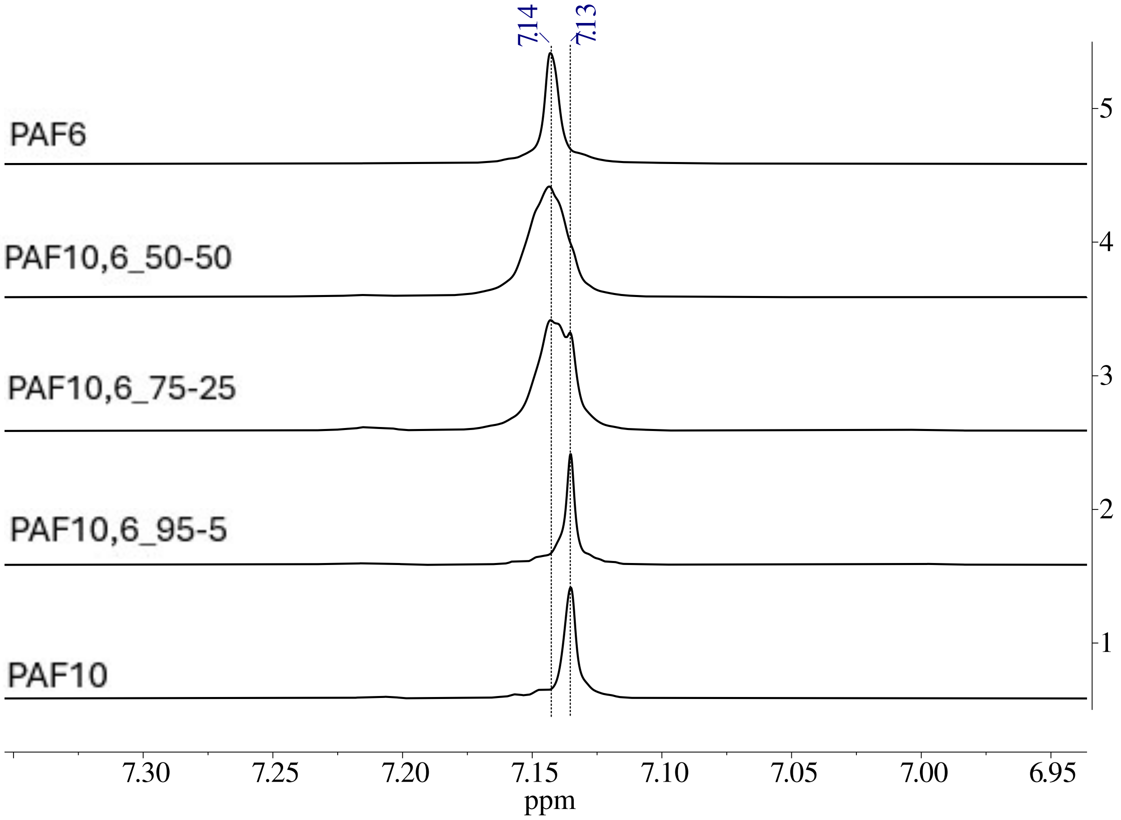

Figure S5: The aromatic region of ^{1}H-NMR spectra of the polyamides, showing the upfield shift of the signals with increasing amount of decanediamine.

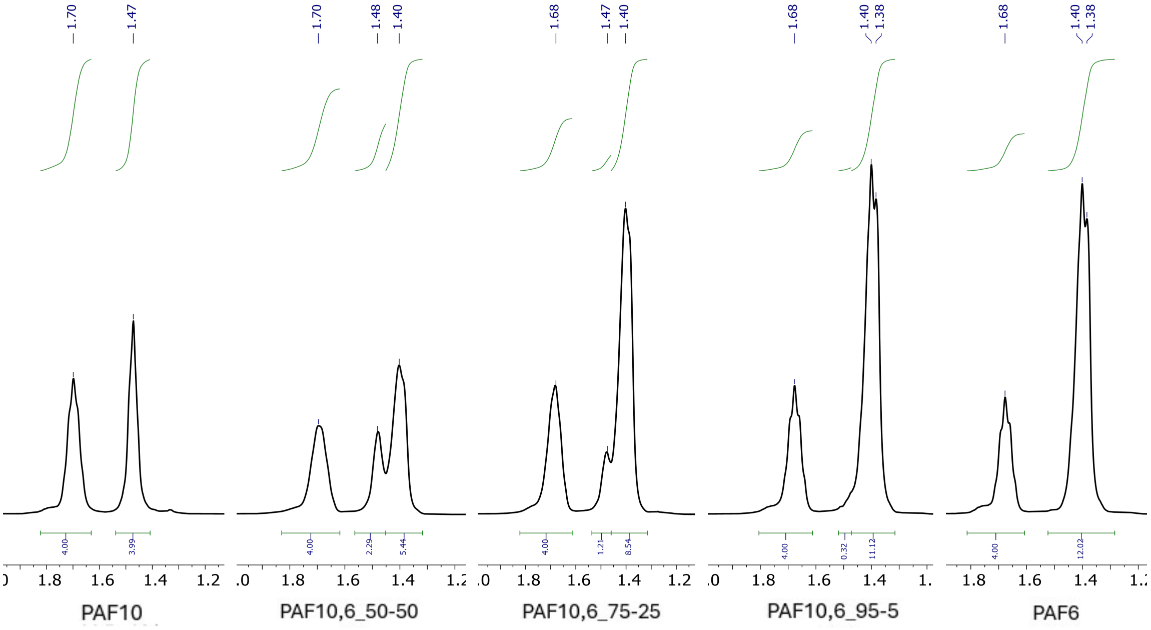

Figure S6: The alkane region of ^{1}H-NMR spectra of the polyamides showing the integrals of the methylene signals used to confirm the decanediamine content of the polyamides and copolyamides.

3 Differential Scanning Calorimetry

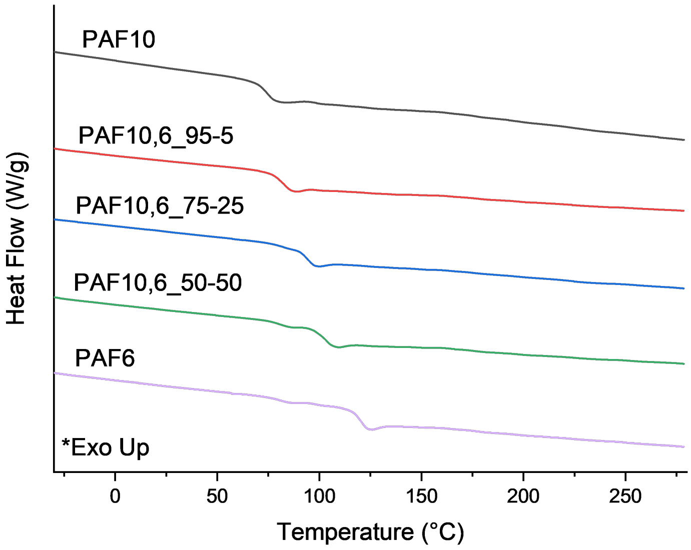

Figure S7: DSC traces from the second heating of the polyamides. Only the glass transition temperature, T_g, can be observed, while no peaks or transitions showing melting or crystallisation can be observed.

4 Thermogravimetric Analysis

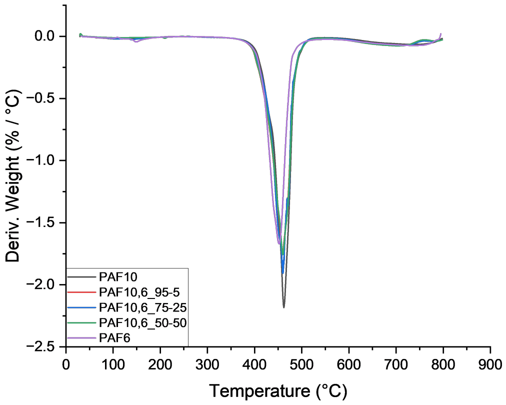

(a)

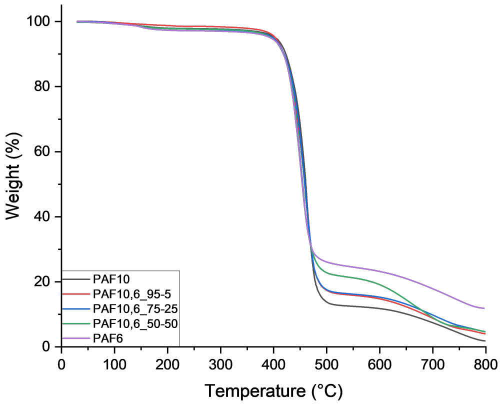

(b)

Figure S8: Thermograms from TGA of the polyamides and copolyamides: (a) Derivative thermogravimetry (DTG), and (b) degradation analysis over temperature.

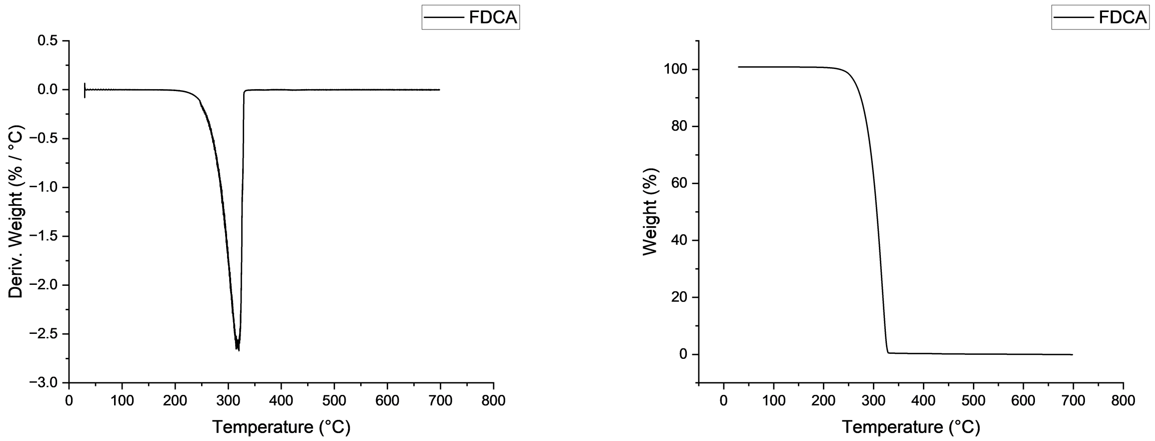

(a) (b)

Figure S9: Thermograms from TGA of FDCA: (a) Derivative thermogravimetry (DTG), and (b) degradation analysis over temperature.

Table S1: Thermal properties and residue at 800 °C of the synthesised polyamides and copolyamides.

Sample	$T_{10\%}$ (°C)	Residue at 800 °C (%)
FDCA	261.4	0
PAF10	423.0	1.9
PAF10,6_95-5	419.1	4.0
PAF10,6_75-25	419.6	4.7
PAF10,6_50-50	418.3	4.7
PAF6	420.1	11.9

5 Dynamic Mechanical Analysis

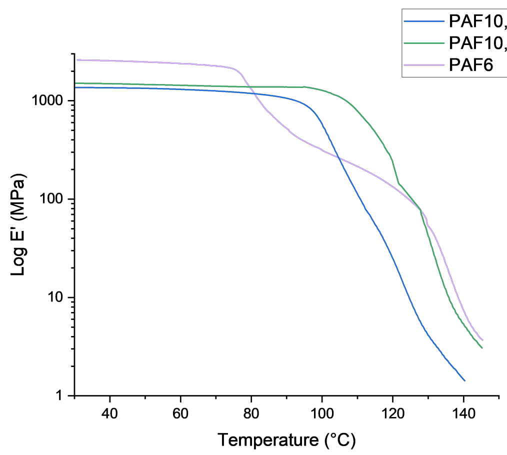

(a)

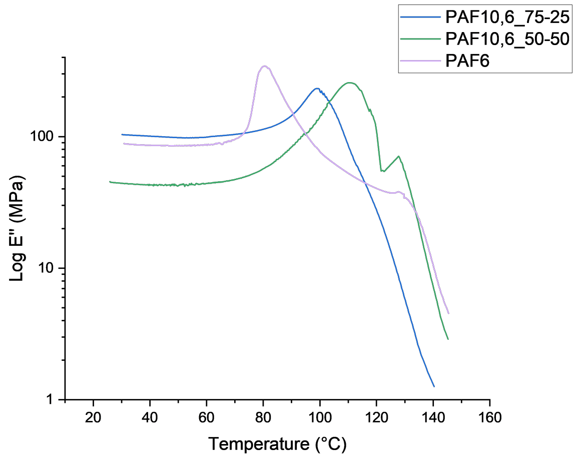

(b)

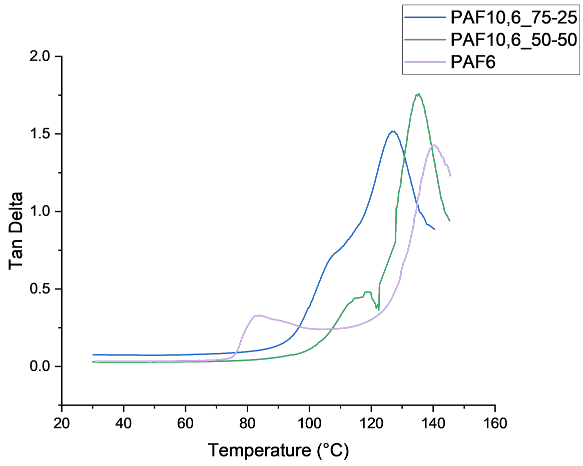

(c)

Figure S10: Viscoelastic properties graphs from DMA analysis: (a) storage modulus (E') vs temperature (b) loss modulus (E") vs. temperature, (c) tan delta vs temperature.
